# High expression of endoplasmic reticulum chaperone grp94 is a novel molecular hallmark of malignant plasma cells in multiple myeloma

**DOI:** 10.1186/s13045-015-0177-6

**Published:** 2015-06-25

**Authors:** Saurabh Chhabra, Sandeep Jain, Caroline Wallace, Feng Hong, Bei Liu

**Affiliations:** Hollings Cancer Center, 86 Jonathan Lucas Street, Charleston, SC 29425 USA; Department of Microbiology and Immunology, Medical University of South Carolina, 86 Jonathan Lucas Street, Charleston, SC 29425 USA; Division of Hematology and Oncology, Department of Medicine, Medical University of South Carolina, Charleston, SC 29425 USA

**Keywords:** Multiple myeloma, Plasma cell, Endoplasmic reticulum, Chaperone, grp94, gp96, Biomarker

## Abstract

**Background:**

Multiple myeloma (MM) is a hematologic malignancy that is characterized by the proliferation of abnormal bone marrow plasma cells (BMPC) and overproduction of immunoglobulin or light chains with evidence of end-organ damage such as bone damage, anemia, hypercalcemia, and renal dysfunction. The pathogenesis of MM is closely linked to dysregulated unfolded protein response (UPR) in the endoplasmic reticulum (ER). Constitutive activation of UPR in mice, as demonstrated by transgenic expression of a master UPR transcription factor XBP1s (a UPR-specific splice variant of X-box binding protein 1), causes myeloma. grp94 (gp96) is a key downstream chaperone in the ER that mediates the UPR as a part of the protein quality control mechanism in the secretory pathway. Our recent study has shown that the persistence of plasma cells as well as the development of myeloma in XBP1s-transgenic mice is critically dependent on grp94. However, the role of grp94 in the initiation and progression of human MM is still unknown.

**Methods:**

The expression level of grp94 in BMPCs was measured by flow cytometry, real-time RT-PCR, and Western blot analysis. We compared the expression levels of grp94 in BMPCs in a spectrum of patients including MM, monoclonal gammopathy of undetermined significance (MGUS), smoldering MM (SMM), as well as non-plasma cell disorders (NPC).

**Results:**

We found that grp94 was highly expressed in malignant plasma cells in patients with MM, but not in BMPCs in patients with MGUS/SMM and NPC. The expression level of grp94 correlated significantly with CD138 expression level. We also found that the grp94 expression level in BMPCs from International Staging System (ISS) stage III MM patients is higher than those in ISS stage I/II MM patients.

**Conclusions:**

grp94 is highly expressed in BMPCs in MM, which correlates with the advanced stage of this disease. Our data demonstrated that grp94 is a novel diagnostic and prognostic biomarker. It also positioned grp94 as a promising therapeutic target for MM.

## Introduction

Despite the introduction of new treatments, multiple myeloma (MM) remains an incurable malignant plasma cell disorder [1–11]. It is the second most common hematologic malignancy, and it accounts for 10 % of all hematological malignancies [12–15]. Each year, over 20,000 new cases are diagnosed in the United States of America [16]. MM is a cytogenetically heterogeneous clonal disorder [13, 17, 18]. It typically evolves from an asymptomatic pre-malignant stage called monoclonal gammopathy of undetermined significance (MGUS) [19–21], to an intermediate asymptomatic smoldering MM (SMM) [22], to eventually symptomatic MM. SMM is a biologically heterogeneous entity [23] which includes patients similar to those with MGUS with a very low rate of progression, as well as those who develop clinically evident end-organ damage within the first 2 years of diagnosis [24, 25]. No single pathological or molecular feature can be used to distinguish MM patients from SMM, who have clonal pre-malignant plasma cells from those with clonal myeloma cells.

Durie and Salmon introduced a staging system in 1975 that used M protein, hemoglobin, calcium, and the number of bone lesions to predict MM cell tumor burden [26]. In the 1980s, serum β2-microglobulin (β2M) was found to be a simple but reliable prognostic marker for staging of MM [27, 28]. Subsequently, albumin [29] and bone marrow plasma cell (BMPC) proliferation indices [30, 31] were found to be useful prognostic factors. The International Staging System (ISS) was devised in 2005 using β2M and serum albumin level, which enabled staging the patients clinically and ascertaining their prognoses [32]. This has been further refined by combining FISH data with ISS [33, 34]. Other types of biomarkers (including serum free light chain (sFLC) ratio and cytogenetic markers) also provide prognostic information on myeloma and for patients with asymptomatic plasma cell disorders [34–37]. Recent studies showed that CD44 expression is significantly higher in plasma cells from persistent/relapsed MM than untreated patients [38]. N-cadherin protein and gene expression are increased in CD38^high^CD138^+^ plasma cells from MM patients, and high expression of N-cadherin correlates with shorter progression-free survival and overall survival comparing with patients with normal N-cadherin level [39]. More recently, it was found that serum miRNA-483-5P is significantly elevated in MM patients, and high expression of miRNA-483-5P is associated with shorter progression-free survival [40]. Insulin-like growth factor binding protein 7 (IGFBP7) expression is associated with the poor prognosis with the absence of myeloma bone disease [41].

The clinical outcome of MM has significantly improved in the last decade due to introduction of the new class of therapeutic agents [42, 43]. Until the 1990s, few advances in treatment of the MM occurred and the median survival of newly diagnosed myeloma patients was 2.5 years [42]. However, beginning in the mid-1990s, with the introduction of high-dose melphalan and autologous hematopoietic cell transplantation (AHCT), survival began to improve [44]. From late 1990s to mid-2000s, the overall median survival increased to nearly 4 years [42]. Further improvement in disease control and survival were made in the mid-2000s with the introduction of highly active agents with mechanisms of action independent of DNA damage [42]. Immunomodulatory drugs (IMiDs) such as thalidomide and lenalidomide and proteasome inhibitors (PI) such as bortezomib are the few examples of these novel agents [45]. However, nearly all MM patients eventually relapse. High rates of relapse suggest that clinically undetectable minimal residual disease persists after treatment, and proliferation of the remaining myeloma cells ultimately results in relapse [18, 46]. Therefore, sensitive and specific early diagnostic and prognostic biomarkers remain necessary for MM.

Molecular chaperone grp94 [47], also known as gp96 [48], endoplasmin [49], ERp99 [50], and HSP90b1 [51], is an endoplasmic reticulum (ER) paralog of HSP90. grp94 is a key downstream chaperone to mediate unfolded protein response (UPR) [52]. UPR is an evolutionally conserved mechanism that maintains protein quality control in the secretory pathway. Accumulation of misfolded proteins in the ER triggers the activation of three well-known pathways: activating transcription factor 6 (ATF6), the double-stranded RNA-activated protein kinase-like ER kinase (PERK), and the spliced form of X-box binding protein 1 (XBP1s). These induce the expression of the major ER heat shock proteins including grp94, grp78, and calreticulin, which together enhance the protein folding machinery [53, 54]. The pathogenesis of MM is closely linked to dysregulated UPR in the ER [55]. Constitutive activation of UPR in mice, as demonstrated by transgenic expression of a master UPR transcription factor XBP1s, causes myeloma [56]. Our recent study has shown that the persistence of plasma cells as well as the development of myeloma in XBP1s-transgenic mice is critically dependent on grp94 [57]. However, the role of grp94 in the initiation and progression of human MM is still unknown. In this study, we examined the expression levels of grp94 in BMPCs from patients with plasma cell disorders and non-plasma cell diseases. We found that high expression of grp94 in BMPCs is a novel molecular hallmark of MM.

## Results

### Patient characteristics

Patient characteristics in this study are summarized in Table 1. The median age was 57 years (range 32–75) for MM patients, 62 years (range 54–72) for MGUS/SMM patients, and 63 years (range 38–76) for NPC patients. The male-to-female ratios were 1.5:1 in MM, 1:2.5 in MGUS/SMM, and 4:1 in NPC. Based on the ISS staging system [32], 4 MM patients were in stage I, 1 patient in stage II, and 13 patients in stage III at the time of diagnosis. The monoclonal component was of IgGκ type in ten patients, IgGλ type in one patient, IgA type in two patients, and light chain disease in seven patients. The median number of prior treatment regimens for MM was two. Three MM patients were treated with autologous hematopoietic cell transplantation. Fifteen NPC patients include 5 diffuse large B cell lymphoma (DLBCL) patients with active disease, 2 acute myelogenous leukemia (AML) patients with active disease, 3 AML patients with no disease, 1 mantle cell lymphoma (MCL) patient with active disease, 1 marginal zone B cell lymphoma (MZL) patient with stage IV active disease, 1 Hodgkin lymphoma (HL) patient with active disease, 1 aplastic anemia (AA) patient, and 1 carcinoma patient with gastrointestinal (GI) primary.

**Table 1:** Clinical characteristics of patients

	MM	MGUS and SMM	NPC
	(*n*)	(*n*)	(*n*)
Total	20	7	15
Male	12	2	12
Female	8	5	3
Range of age (years)	32–75	54–72	38–76
ISS			
Stage I	4		
Stage II	1		
Stage III	13		
Type of monoclonal component			
IgG, κ	10	3	
IgG, λ	1	2	
IgA	2	1	
Light chain	7	1	
Non-secretory	0		
β2 microglobulin	5.8		
Median number of prior regimens	2		
Auto HSCT	3		
Prior bortezomib	14		
Prior lenalidomide	9		
Prior thalidomide	2		

### grp94 is preferentially expressed in plasma cells

**Fig. 1:**
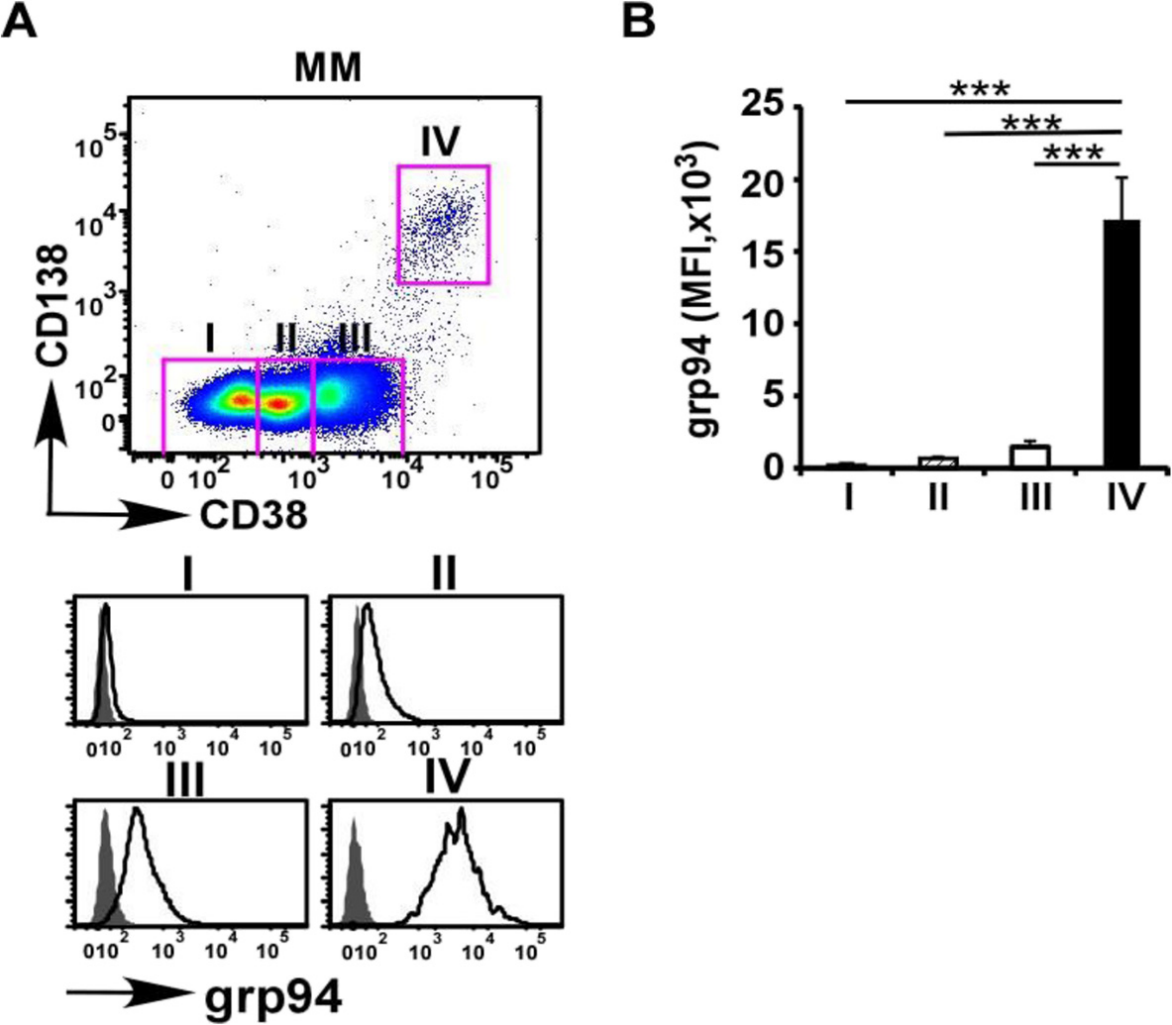
grp94 is preferentially expressed on plasma cells. **a** Human bone marrow cells were analyzed and representative FACS plots of different populations in MM. *Histograms* show the intracellular staining of grp94 on different populations (*open histogram* indicates grp94; *shaded histogram*, isotype control). Population *I* CD38^−^CD138^−^ cells (non-B cells), population *II* CD38^low^CD138^−^ cells (naïve B cells), population *III* CD38^int^CD138^−^ cells (pro- and pre-B cells), population *IV* CD38^high^CD138^+^ cells (plasma cells). **b** Quantification of grp94 expression in different populations of bone marrow from the patients with MM. *Error bars* indicate standard error of the mean. ***p < 0.001

We first examined the protein expression level of grp94 on various cellular populations in the bone marrow (BM) using flow cytometry after intracellular staining with a highly specific monoclonal antibody against grp94. Remarkably, although grp94 is thought to be constitutively expressed by all nucleated cells [52], we found that the expression level of grp94 in human BM cells in MM is extremely heterogeneous. Non-B cells (population I: CD38^−^CD138^−^ cells), which are mostly myeloid cells, express grp94 at very low level. By comparison, plasma cells (population IV: CD38^high^CD138^+^ cells) express the highest level of grp94, followed by pro- and pre-B cells (population III: CD38^int^CD138^−^ cells) and naïve B cells (population II: CD38^low^CD138^−^ cells) (Fig. 1a, b).

**Fig. 2:**
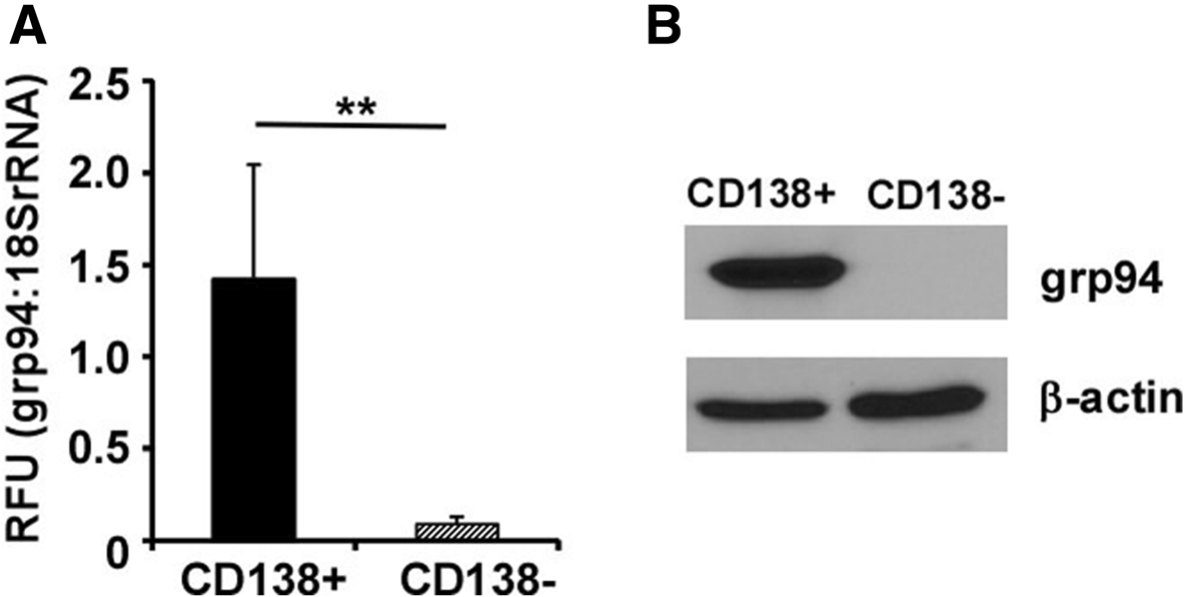
grp94 is highly expressed in plasma cells. **a** Q-RT-PCR analysis of grp94 mRNA in CD138^+^ plasma cells and CD138^−^ non-plasma cells. 18S ribosomal RNA was used as an internal control. The quantity of transcripts for respective genes was expressed as a relative fluorescence unit (*RFU*), calculated based on the number of cycles to reach the threshold of detection (Ct) using the formula of 2^ΔCt^ (Ct _interest_ − Ct _18S rRNA_). **b** Western blot for grp94 of CD138^+^ plasma cells and CD138^−^ non-plasma cells. β-actin was blotted to indicate equal loading of cell lysates

We next corroborated our findings by determining the mRNA expression level of grp94 in BMPCs in MM patients by real-time RT-PCR as well as Western blot. We isolated CD138^+^ PCs and CD138^−^ non-plasma cells from BM cells from MM patients using magnetic beads, followed by purification of total mRNA and real-time RT-PCR using primers specific for human grp94 cDNA. 18S ribosomal RNA was used as an internal control. We found that grp94 transcript level in CD138^+^ BMPCs was more than 15-fold higher than CD138^−^ cells (Fig. 2a). Consistently, the significant high expression level of grp94 in CD138^+^ PCs was also confirmed by Western blot (Fig. 2b).

### grp94 is highly expressed in malignant plasma cells in MM

**Fig. 3:**
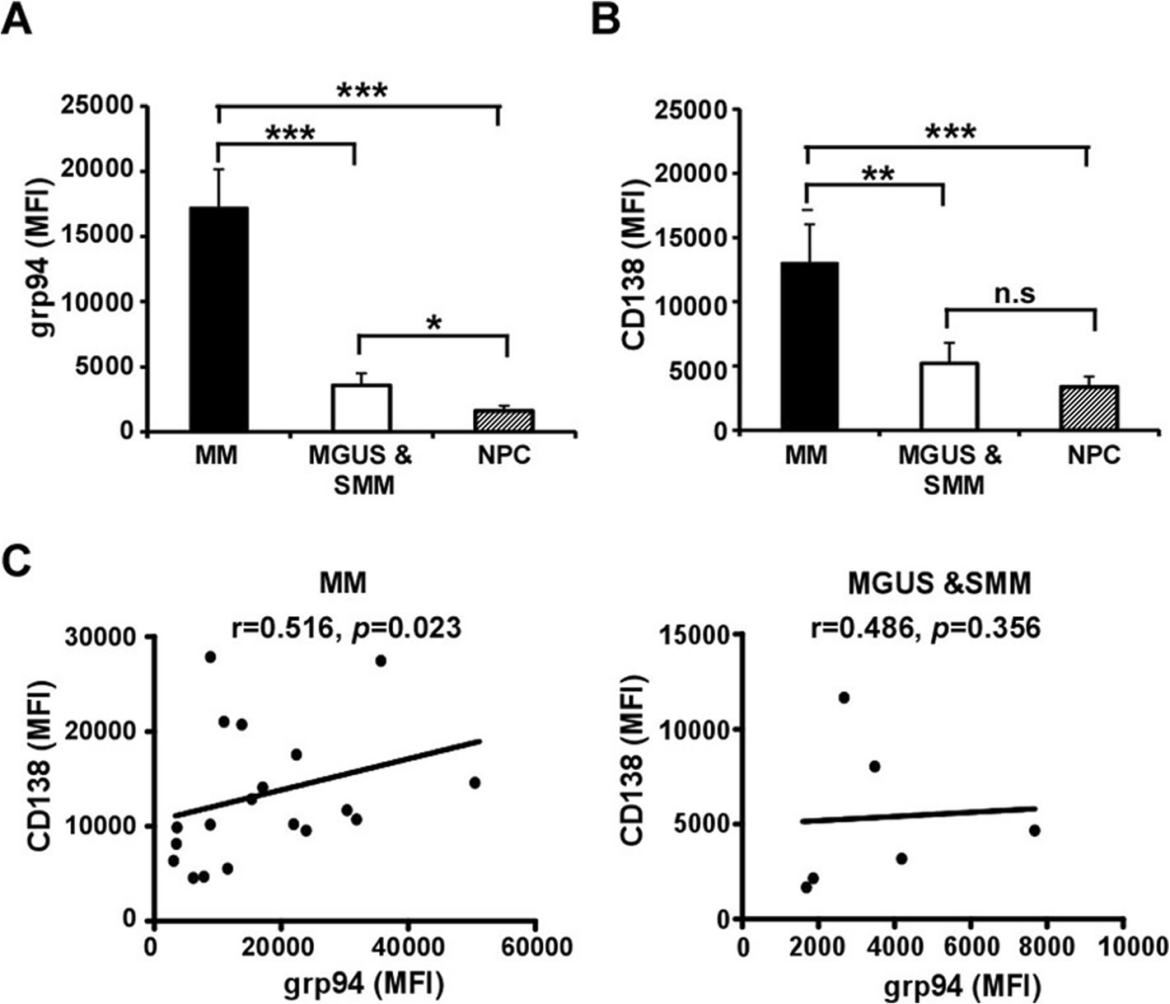
grp94 is highly expressed on malignant plasma cells in MM. **a** Quantification of grp94 expression in the plasma cells from the patients with MM, MGUS/SMM, and NPC. *Error bars* indicate standard error of the mean. *p < 0.05, **p < 0.01, ***p < 0.001. **b** Quantification of CD138 expression in the plasma cells from the patients with MM, MGUS/SMM, and NPC. *Error bars* indicate standard error of the mean. **p < 0.01, ***p < 0.001. **c** Correlations between grp94 expression and CD138 expression in plasma cells from patients with MM (*left panel*) and patients with MGUS/SMM (*right panel*)

We next determined if the high level of grp94 expression is unique to BMPCs of MM patients by broadening our analysis to patients with MGUS/SMM as well as NPC. As expected, BMPCs from MM patients expressed a significantly higher level of grp94, compared with BMPCs from patients with MGUS/SMM and NPC (Fig. 3a). CD138 (syndecan-1) is a specific marker of human plasma cells [58]. We found indeed that the expression of grp94 paralleled with CD138 level (Fig. 3c, left panel) in MM patients. However, while grp94 expression level separated out MGUS/SMM from NPCs, CD138 level failed to do so (Fig. 3a, b), demonstrating that grp94 expression is a better diagnostic marker than CD138 for malignant plasma cell disorder.

### Higher expression of grp94 is associated with worse disease in MM

**Fig. 4:**
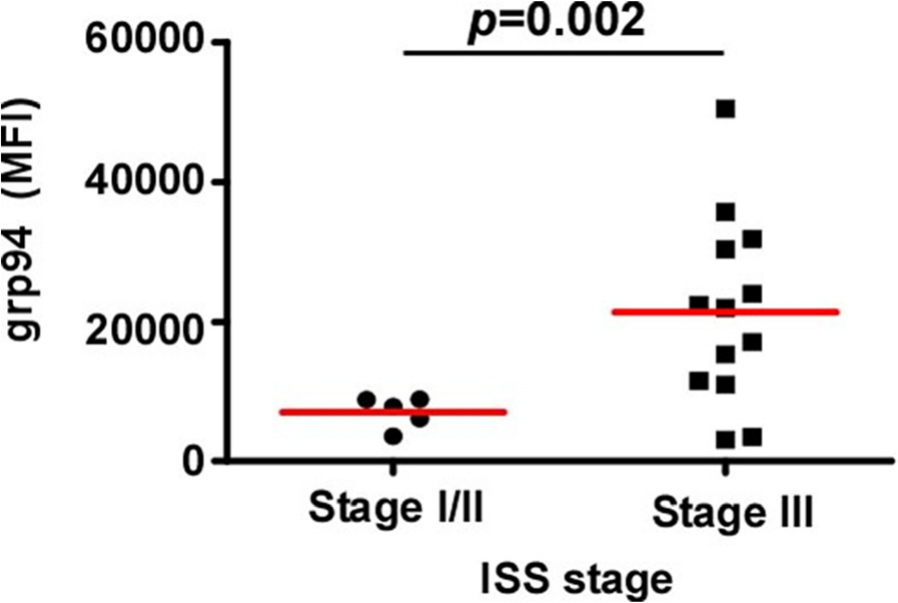
Higher expression of grp94 is associated with worse disease in MM. Compare grp94 expression level in malignant plasma cells from MM patients with different ISS stages. The grp94 expression level is significantly higher in malignant plasma cells from stage III MM patients than that from stage I/II MM patients. (*p* = 0.002)

We next examined if the grp94 expression in malignant plasma cells in MM patients has any prognostic significance, by comparing grp94 expression level in BMPCs with the ISS staging status [32]. We found that the expression level of grp94 correlated with the clinical outcomes of MM patients. grp94 expression is significantly elevated in plasma cells from stage III MM patients compared to the ones at the stage I/II (Fig. 4). Collectively, we have demonstrated that high level of grp94 expression is not only a molecular hallmark unique to BMPCs of MM patients but it also predicts poorer clinical outcomes in MM patients.

## Discussion

MM is characterized by the infiltration of bone marrow with clonal plasma cells that secrete a monoclonal protein in the majority of patients [59]. The hallmark of the pathology is the overproduction of a secreted protein by a malignant plasma cell population, and UPR plays critical roles in the pathogenesis of MM. grp94 is a key downstream chaperone in the ER and mediates UPR, but its roles in human MM was unexplored [52, 60]. In this study, we discovered for the first time that human plasma cells express significantly high level of grp94, when compared with other cellular populations, indicating that the plasma cells are under active ER stress. More importantly, we found that grp94 is highly expressed in malignant plasma cells in MM, when compared with MGUS/SMM and NPC. Our observation is consistent with the notion that grp94 is critical for malignant plasma cell survival and persistence [57], but not for benign plasma cells [61]. CD138 is commonly expressed by plasma cells, but it is not a unique diagnostic marker for MM [58]. A recent study showed that the serum level of CD138 was significantly higher in active MM than that in MGUS [62]. Consistent to this study, we found that CD138 is highly expressed on plasma cells from the MM patients, when compared with patients with MGUS/SMM and NPC. We also observed that grp94 expression significantly correlated with the CD138 expression in plasma cells from patients with MM, but not with MGUS/SMM. Further studies with a larger cohort of patients shall solidify this conclusion. Interestingly, despite a small sample size, we discovered that the grp94 expression in malignant plasma cells likely has prognostic significance. Its level is significantly elevated in plasma cells from the ISS stage III MM patients in comparison with the stage I/II. A future perspective study is warranted to further establish both the diagnostic and prognostic value of BMPC-intrinsic grp94 expression in MM and other plasma cell disorders.

Among many client proteins chaperoned by grp94 [52, 61, 63, 64], Wnt co-receptor low-density lipoprotein receptor-related protein 6 (LRP6) for canonical Wnt signaling depends exclusively on grp94 for folding [57, 65]. Our study thus may add significantly to the emerging roles of Wnt signaling in MM [66–68]*.* Human MM cells appear to have evidence of active Wnt signaling by overexpressing β-catenin which promotes proliferation of malignant cells [69]. Blocking β-catenin with small-molecule inhibitors, AV-65 [68] or PKF115-584 [66, 67], specifically inhibits MM cell proliferation. Our previous study showed that in the absence of grp94, MM cells undergo mitotic catastrophe and apoptosis which correlated with decreased expression of survivin, a downstream target molecule of Wnt signaling [57].

Finally, grp94 might promote the pathogenesis of MM through folding other client proteins such as integrins, IGF, and Toll-like receptors [61, 63, 64]. Intriguingly, grp94-selective inhibitors are already in early development [70, 71]. Our study may therefore pave a way for developing grp94-targeted strategy for the treatment of MM.

## Conclusions

In summary, we found that grp94 is highly expressed in malignant plasma cells in multiple myeloma. The higher level of grp94 is significantly associated with worse clinical stage in this disease. Our data demonstrated that grp94 is not only a specific diagnostic and prognostic biomarker but also a therapeutic target in multiple myeloma.

## Methods

### Study subjects

Forty-two patients at the Hollings Cancer Center, Medical University of South Carolina, were enrolled in this study. Bone marrow samples were taken from 20 patients with MM, 7 patients diagnosed with MGUS or SMM, and 15 patients with NPC, which include DLBCL, AML, MCL, MZL, HL, AA, and GI primary carcinoma. This study was conducted in accordance with the ethical guidelines and was approved by the Institutional Review Board of the Medical University of South Carolina. Informed consent was obtained from all patients.

### Reagents

Antibodies used for flow cytometry were obtained from BD Biosciences (Mountain View, CA) and eBioscience (San Diego, CA). Antibodies against grp94 Ab (9G10) was bought from Enzo Life Sciences, Inc. (Farmingdale, NY), and β-actin Ab (AC-74) was purchased from Sigma-Aldrich (St Louis, MO). All other chemicals were obtained from Sigma-Aldrich (St Louis, MO) and Fisher Scientific (Pittsburgh, PA).

### Isolation of bone marrow cells

Add Histopaque-1077 to a 15-mL conical centrifuge tube and warm to room temperature. Carefully add anticoagulated bone marrow aspirate onto the Histopaque-1077 at 1:1 ratio (Sigma-Aldrich, St Louis, MO). Centrifuge at 400 *g* for 30 min at room temperature. After centrifugation, aspirate the upper layer with a Pasteur pipette to within 0.5 cm of the opaque interface containing bone marrow cells. Discard upper layer and transfer the opaque interface into a clean conical centrifuge tube. Wash the cells by adding 10 mL PBS and centrifuge at 1500 rpm for 5 min.

### Flow cytometry

Surface staining of cells and flow cytometry were done as described [64, 72, 73]. To stain grp94 intracellularly, cells were fixed in 4 % paraformaldehyde and permeablized in ice-cold methanol. Cells were acquired on FACSVerse (Becton Dickinson, Franklin Lakes, NJ), and results were analyzed with FloJo software (Tree Star, Ashland, OR).

### Quantitative RT-PCR

CD138^+^ and CD138^−^ cells were isolated from BM by using human CD138 positive selection kit (StemCell Technologies Inc., Vancouver, Canada). Total RNA was extracted with the RNeasy Mini kit according to the manufacturer’s protocol (Qiagen, Valencia, CA). First-strand cDNA was synthesized with Superscript II (Invitrogen, Carlsbad, CA). cDNA was quantified by Q-PCR with Bio-Rad CFX Connect Real-Time System. Q-PCR data were analyzed with the corresponding software, and the number of PCR cycles to reach the threshold of detection (CT) was calculated. Samples were run in duplicates. 18S ribosomal RNA (18S rRNA) was used as an internal control. The primer sequences are as follows. Human grp94F, GCTTCGGTCAGGGTATCTTT; human grp94R, AGGCTCTTCTTCCACCTTTG; 18S rRNA F, CGGCTACCACATCCAAGGAA; 18S rRNA R, GCTGGAATTACCGCGGCT.

### Protein extraction and Western blot

Protein extraction and immunoblot were performed as described previously [74]. Briefly, cells were washed three times with ice-cold PBS and lysed in radioimmunoprecipitation assay (RIPA) lysis buffer (0.01 M sodium phosphate, pH 7.2, 150 mM NaCl, 2 mM EDTA, 1 % NP-40, 1 % sodium deoxycholate, 0.1 % SDS, 2 mM AEBSF, 130 mM bestatin, 14 mM E-64, 0.3 mM aprotinin, and 1 mM leupeptin). Total cell lysates was resolved on denaturing and reducing 10 % SDS-PAGE, and the proteins were transferred from the gel onto Immobilon-P membranes. The membrane was blocked with 5 % nonfat milk in PBS and then incubated with different Abs, followed by incubation with HRP-conjugated secondary Ab. Protein bands were visualized by using enhanced chemiluminescent substrate (Pierce, Rockford, IL).

### Statistical analysis

Error bars represent the standard error of the mean (SEM). Independent-samples *t* tests or ANOVA were used to compare variables between different groups. Correlations between variables were assessed using Pearson correlation analysis. All statistical analyses were performed using Prism 5 software. Values of *P* less than 0.05 were considered to represent statistically significant differences.

## Abbreviations

MM: multiple myeloma
MGUS: monoclonal gammopathy of undetermined significance
SMM: smoldering multiple myeloma
NPC: non-plasma cell disorder
ER: endoplasmic reticulum
HSP: heat shock protein
UPR: unfolded protein response
ATF6: activating transcription factor 6
PERK: the double-stranded RNA-activated protein kinase-like ER kinase
XBP1s: spliced form of X-box binding protein 1
BM: bone marrow
